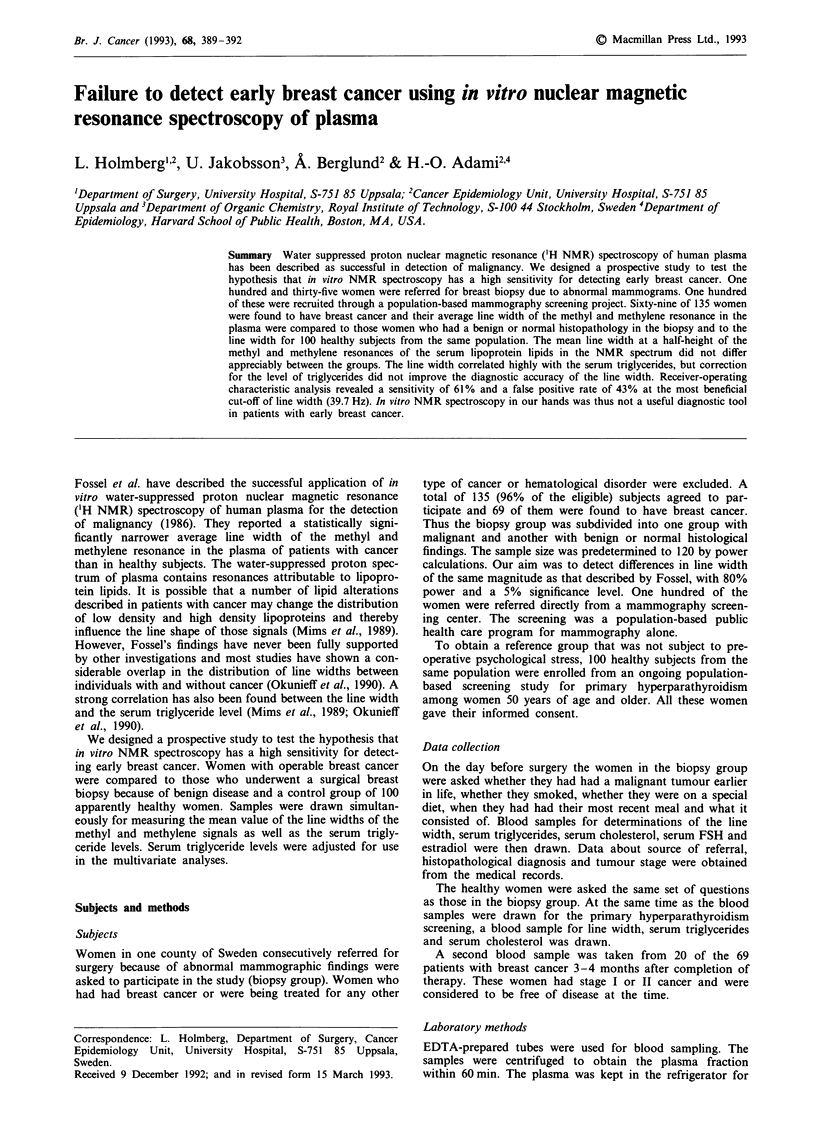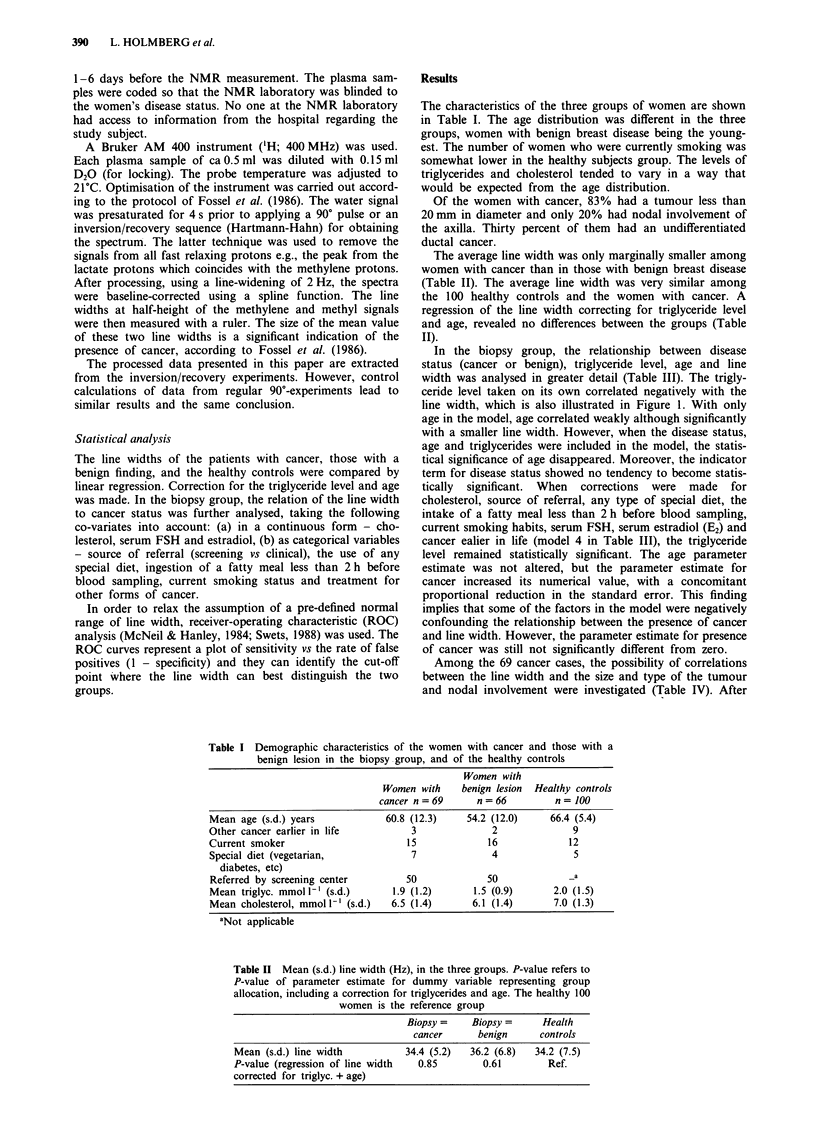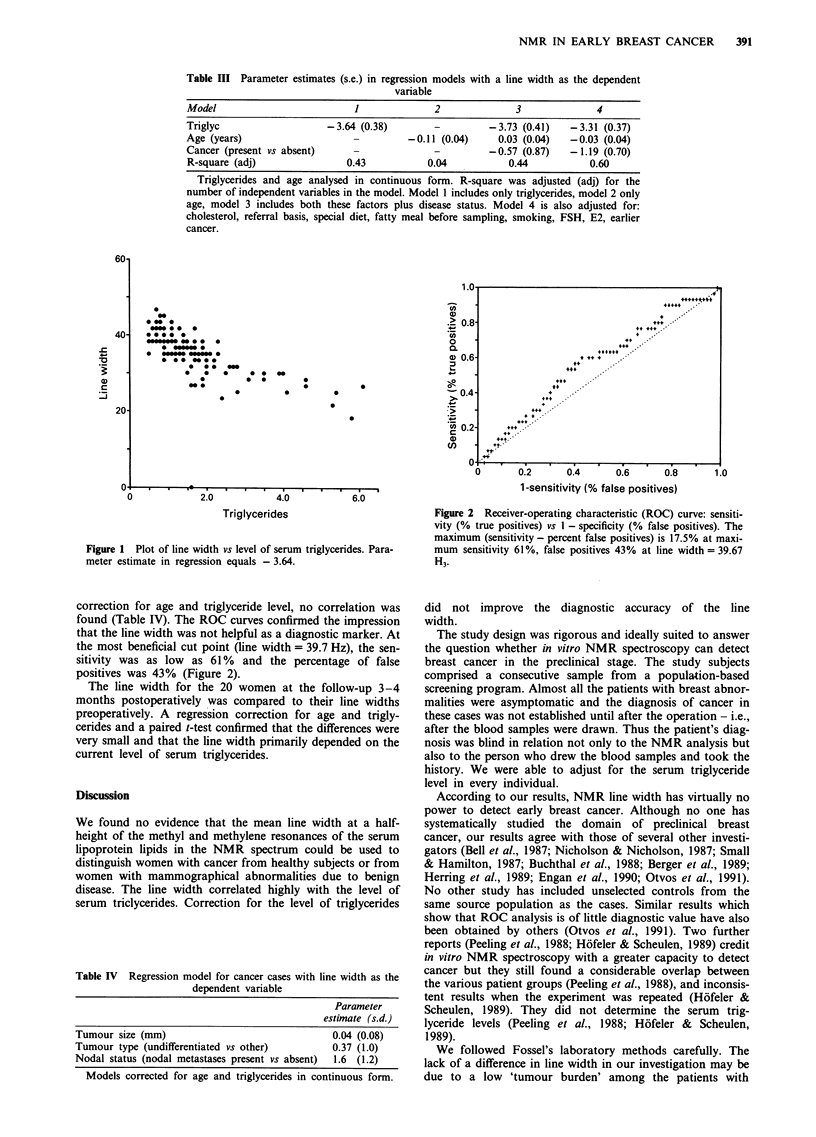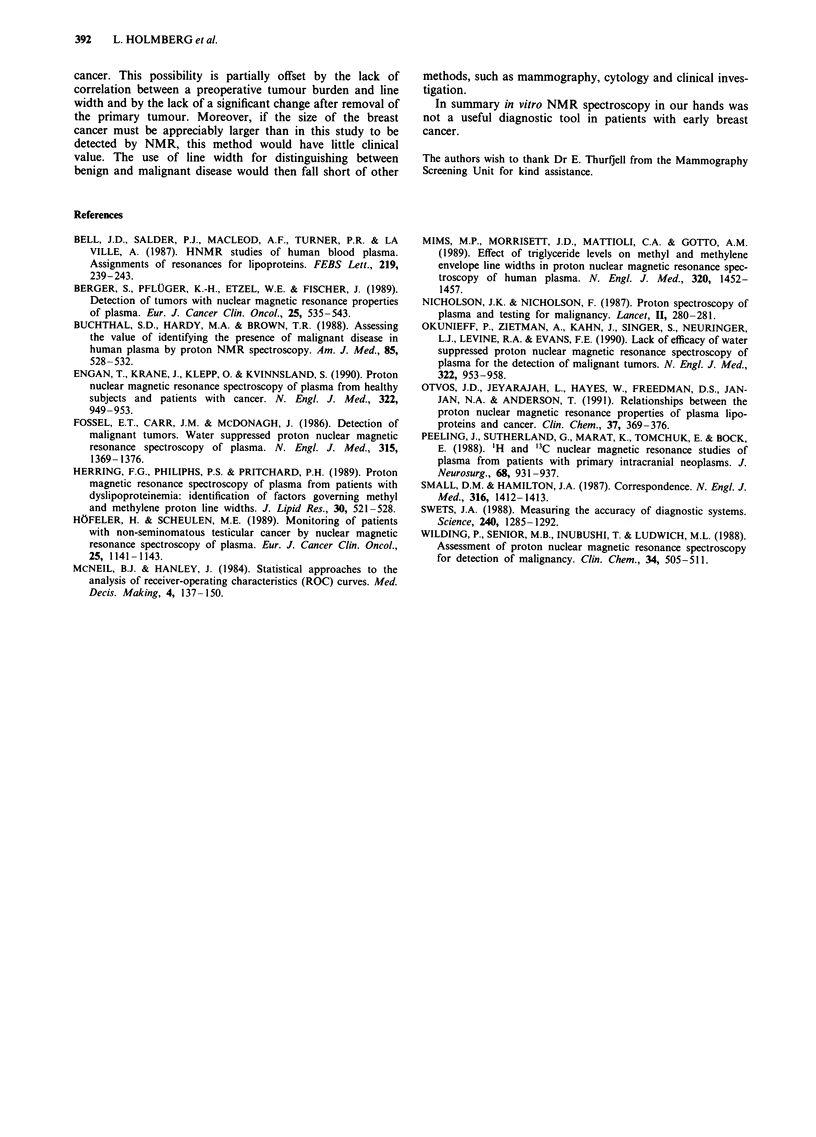# Failure to detect early breast cancer using in vitro nuclear magnetic resonance spectroscopy of plasma.

**DOI:** 10.1038/bjc.1993.346

**Published:** 1993-08

**Authors:** L. Holmberg, U. Jakobsson, A. Berglund, H. O. Adami

**Affiliations:** Department of Surgery, University Hospital, Uppsala, Sweden.

## Abstract

Water suppressed proton nuclear magnetic resonance (1H NMR) spectroscopy of human plasma has been described as successful in detection of malignancy. We designed a prospective study to test the hypothesis that in vitro NMR spectroscopy has a high sensitivity for detecting early breast cancer. One hundred and thirty-five women were referred for breast biopsy due to abnormal mammograms. One hundred of these were recruited through a population-based mammography screening project. Sixty-nine of 135 women were found to have breast cancer and their average line width of the methyl and methylene resonance in the plasma were compared to those women who had a benign or normal histopathology in the biopsy and to the line width for 100 healthy subjects from the same population. The mean line width at a half-height of the methyl and methylene resonances of the serum lipoprotein lipids in the NMR spectrum did not differ appreciably between the groups. The line width correlated highly with the serum triglycerides, but correction for the level of triglycerides did not improve the diagnostic accuracy of the line width. Receiver-operating characteristic analysis revealed a sensitivity of 61% and a false positive rate of 43% at the most beneficial cut-off of line width (39.7 Hz). In vitro NMR spectroscopy in our hands was thus not a useful diagnostic tool in patients with early breast cancer.


Br. J. Cancer (1993), 68, 389-392 © Macmillan Press Ltd., 1993

### Failure to detect early breast cancer using *in vitro* nuclear magnetic resonance spectroscopy of plasma

L. Holmberg[1,2], U. Jakobsson[3], Å. Berglund[2] & H.-O. Adami[2,4]

[1]*Department of Surgery, University Hospital, S-751 85 Uppsala;* [2]*Cancer Epidemiology Unit, University Hospital, S-751 85 Uppsala and* [3]*Department of Organic Chemistry, Royal Institute of Technology, S-100 44 Stockholm, Sweden* [4]*Department of Epidemiology, Harvard School of Public Health, Boston, MA, USA.*

**Summary** Water suppressed proton nuclear magnetic resonance ($^1$H NMR) spectroscopy of human plasma has been described as successful in detection of malignancy. We designed a prospective study to test the hypothesis that *in vitro* NMR spectroscopy has a high sensitivity for detecting early breast cancer. One hundred and thirty-five women were referred for breast biopsy due to abnormal mammograms. One hundred of these were recruited through a population-based mammography screening project. Sixty-nine of 135 women were found to have breast cancer and their average line width of the methyl and methylene resonance in the plasma were compared to those women who had a benign or normal histopathology in the biopsy and to the line width for 100 healthy subjects from the same population. The mean line width at a half-height of the methyl and methylene resonances of the serum lipoprotein lipids in the NMR spectrum did not differ appreciably between the groups. The line width correlated highly with the serum triglycerides, but correction for the level of triglycerides did not improve the diagnostic accuracy of the line width. Receiver-operating characteristic analysis revealed a sensitivity of 61% and a false positive rate of 43% at the most beneficial cut-off of line width (39.7 Hz). *In vitro* NMR spectroscopy in our hands was thus not a useful diagnostic tool in patients with early breast cancer.

Fossel *et al.* have described the successful application of *in vitro* water-suppressed proton nuclear magnetic resonance ($^1$H NMR) spectroscopy of human plasma for the detection of malignancy (1986). They reported a statistically significantly narrower average line width of the methyl and methylene resonance in the plasma of patients with cancer than in healthy subjects. The water-suppressed proton spectrum of plasma contains resonances attributable to lipoprotein lipids. It is possible that a number of lipid alterations described in patients with cancer may change the distribution of low density and high density lipoproteins and thereby influence the line shape of those signals (Mims *et al.*, 1989). However, Fossel's findings have never been fully supported by other investigations and most studies have shown a considerable overlap in the distribution of line widths between individuals with and without cancer (Okunieff *et al.*, 1990). A strong correlation has also been found between the line width and the serum triglyceride level (Mims *et al.*, 1989; Okunieff *et al.*, 1990).

We designed a prospective study to test the hypothesis that *in vitro* NMR spectroscopy has a high sensitivity for detecting early breast cancer. Women with operable breast cancer were compared to those who underwent a surgical breast biopsy because of benign disease and a control group of 100 apparently healthy women. Samples were drawn simultaneously for measuring the mean value of the line widths of the methyl and methylene signals as well as the serum triglyceride levels. Serum triglyceride levels were adjusted for use in the multivariate analyses.

### Subjects and methods

### *Subjects*

Women in one county of Sweden consecutively referred for surgery because of abnormal mammographic findings were asked to participate in the study (biopsy group). Women who had had breast cancer or were being treated for any other type of cancer or hematological disorder were excluded. A total of 135 (96% of the eligible) subjects agreed to participate and 69 of them were found to have breast cancer. Thus the biopsy group was subdivided into one group with malignant and another with benign or normal histological findings. The sample size was predetermined to 120 by power calculations. Our aim was to detect differences in line width of the same magnitude as that described by Fossel, with 80% power and a 5% significance level. One hundred of the women were referred directly from a mammography screening center. The screening was a population-based public health care program for mammography alone.

To obtain a reference group that was not subject to preoperative psychological stress, 100 healthy subjects from the same population were enrolled from an ongoing population-based screening study for primary hyperparathyroidism among women 50 years of age and older. All these women gave their informed consent.

### *Data collection*

On the day before surgery the women in the biopsy group were asked whether they had had a malignant tumour earlier in life, whether they smoked, whether they were on a special diet, when they had had their most recent meal and what it consisted of. Blood samples for determinations of the line width, serum triglycerides, serum cholesterol, serum FSH and estradiol were then drawn. Data about source of referral, histopathological diagnosis and tumour stage were obtained from the medical records.

The healthy women were asked the same set of questions as those in the biopsy group. At the same time as the blood samples were drawn for the primary hyperparathyroidism screening, a blood sample for line width, serum triglycerides and serum cholesterol was drawn.

A second blood sample was taken from 20 of the 69 patients with breast cancer 3–4 months after completion of therapy. These women had stage I or II cancer and were considered to be free of disease at the time.

### *Laboratory methods*

EDTA-prepared tubes were used for blood sampling. The samples were centrifuged to obtain the plasma fraction within 60 min. The plasma was kept in the refrigerator for

Correspondence: L. Holmberg, Department of Surgery, Cancer Epidemiology Unit, University Hospital, S-751 85 Uppsala, Sweden.
Received 9 December 1992; and in revised form 15 March 1993.

1–6 days before the NMR measurement. The plasma samples were coded so that the NMR laboratory was blinded to the women's disease status. No one at the NMR laboratory had access to information from the hospital regarding the study subject.

A Bruker AM 400 instrument ($^1$H; 400 MHz) was used. Each plasma sample of ca 0.5 ml was diluted with 0.15 ml $D_2O$ (for locking). The probe temperature was adjusted to 21°C. Optimisation of the instrument was carried out according to the protocol of Fossel *et al.* (1986). The water signal was presaturated for 4 s prior to applying a 90° pulse or an inversion/recovery sequence (Hartmann-Hahn) for obtaining the spectrum. The latter technique was used to remove the signals from all fast relaxing protons e.g., the peak from the lactate protons which coincides with the methylene protons. After processing, using a line-widening of 2 Hz, the spectra were baseline-corrected using a spline function. The line widths at half-height of the methylene and methyl signals were then measured with a ruler. The size of the mean value of these two line widths is a significant indication of the presence of cancer, according to Fossel *et al.* (1986).

The processed data presented in this paper are extracted from the inversion/recovery experiments. However, control calculations of data from regular 90°-experiments lead to similar results and the same conclusion.

### *Statistical analysis*

The line widths of the patients with cancer, those with a benign finding, and the healthy controls were compared by linear regression. Correction for the triglyceride level and age was made. In the biopsy group, the relation of the line width to cancer status was further analysed, taking the following co-variates into account: (a) in a continuous form – cholesterol, serum FSH and estradiol, (b) as categorical variables – source of referral (screening *vs* clinical), the use of any special diet, ingestion of a fatty meal less than 2 h before blood sampling, current smoking status and treatment for other forms of cancer.

In order to relax the assumption of a pre-defined normal range of line width, receiver-operating characteristic (ROC) analysis (McNeil & Hanley, 1984; Swets, 1988) was used. The ROC curves represent a plot of sensitivity *vs* the rate of false positives (1 – specificity) and they can identify the cut-off point where the line width can best distinguish the two groups.

### Results

The characteristics of the three groups of women are shown in Table I. The age distribution was different in the three groups, women with benign breast disease being the youngest. The number of women who were currently smoking was somewhat lower in the healthy subjects group. The levels of triglycerides and cholesterol tended to vary in a way that would be expected from the age distribution.

Of the women with cancer, 83% had a tumour less than 20 mm in diameter and only 20% had nodal involvement of the axilla. Thirty percent of them had an undifferentiated ductal cancer.

The average line width was only marginally smaller among women with cancer than in those with benign breast disease (Table II). The average line width was very similar among the 100 healthy controls and the women with cancer. A regression of the line width correcting for triglyceride level and age, revealed no differences between the groups (Table II).

In the biopsy group, the relationship between disease status (cancer or benign), triglyceride level, age and line width was analysed in greater detail (Table III). The triglyceride level taken on its own correlated negatively with the line width, which is also illustrated in Figure 1. With only age in the model, age correlated weakly although significantly with a smaller line width. However, when the disease status, age and triglycerides were included in the model, the statistical significance of age disappeared. Moreover, the indicator term for disease status showed no tendency to become statistically significant. When corrections were made for cholesterol, source of referral, any type of special diet, the intake of a fatty meal less than 2 h before blood sampling, current smoking habits, serum FSH, serum estradiol ($E_2$) and cancer ealier in life (model 4 in Table III), the triglyceride level remained statistically significant. The age parameter estimate was not altered, but the parameter estimate for cancer increased its numerical value, with a concomitant proportional reduction in the standard error. This finding implies that some of the factors in the model were negatively confounding the relationship between the presence of cancer and line width. However, the parameter estimate for presence of cancer was still not significantly different from zero.

Among the 69 cancer cases, the possibility of correlations between the line width and the size and type of the tumour and nodal involvement were investigated (Table IV). After

**Table I** Demographic characteristics of the women with cancer and those with a benign lesion in the biopsy group, and of the healthy controls

| | *Women with cancer n = 69* | *Women with benign lesion n = 66* | *Healthy controls n = 100* |
|---|---|---|---|
| Mean age (s.d.) years | 60.8 (12.3) | 54.2 (12.0) | 66.4 (5.4) |
| Other cancer earlier in life | 3 | 2 | 9 |
| Current smoker | 15 | 16 | 12 |
| Special diet (vegetarian, diabetes, etc) | 7 | 4 | 5 |
| Referred by screening center | 50 | 50 | –[a] |
| Mean triglyc. mmol $l^{-1}$ (s.d.) | 1.9 (1.2) | 1.5 (0.9) | 2.0 (1.5) |
| Mean cholesterol, mmol $l^{-1}$ (s.d.) | 6.5 (1.4) | 6.1 (1.4) | 7.0 (1.3) |

[a]Not applicable

**Table II** Mean (s.d.) line width (Hz), in the three groups. *P*-value refers to *P*-value of parameter estimate for dummy variable representing group allocation, including a correction for triglycerides and age. The healthy 100 women is the reference group

| | *Biopsy = cancer* | *Biopsy = benign* | *Health controls* |
|---|---|---|---|
| Mean (s.d.) line width | 34.4 (5.2) | 36.2 (6.8) | 34.2 (7.5) |
| *P*-value (regression of line width corrected for triglyc. + age) | 0.85 | 0.61 | Ref. |

**Table III** Parameter estimates (s.e.) in regression models with a line width as the dependent variable

| *Model* | *1* | *2* | *3* | *4* |
|---|---|---|---|---|
| Triglyc | −3.64 (0.38) | – | −3.73 (0.41) | −3.31 (0.37) |
| Age (years) | – | −0.11 (0.04) | 0.03 (0.04) | −0.03 (0.04) |
| Cancer (present *vs* absent) | – | – | −0.57 (0.87) | −1.19 (0.70) |
| R-square (adj) | 0.43 | 0.04 | 0.44 | 0.60 |

Triglycerides and age analysed in continuous form. R-square was adjusted (adj) for the number of independent variables in the model. Model 1 includes only triglycerides, model 2 only age, model 3 includes both these factors plus disease status. Model 4 is also adjusted for: cholesterol, referral basis, special diet, fatty meal before sampling, smoking, FSH, E2, earlier cancer.

**Figure 1** Plot of line width *vs* level of serum triglycerides. Parameter estimate in regression equals − 3.64.

**Figure 2** Receiver-operating characteristic (ROC) curve: sensitivity (% true positives) *vs* 1 − specificity (% false positives). The maximum (sensitivity − percent false positives) is 17.5% at maximum sensitivity 61%, false positives 43% at line width = 39.67 $H_3$.

correction for age and triglyceride level, no correlation was found (Table IV). The ROC curves confirmed the impression that the line width was not helpful as a diagnostic marker. At the most beneficial cut point (line width = 39.7 Hz), the sensitivity was as low as 61% and the percentage of false positives was 43% (Figure 2).

The line width for the 20 women at the follow-up 3–4 months postoperatively was compared to their line widths preoperatively. A regression correction for age and triglycerides and a paired *t*-test confirmed that the differences were very small and that the line width primarily depended on the current level of serum triglycerides.

### Discussion

We found no evidence that the mean line width at a half-height of the methyl and methylene resonances of the serum lipoprotein lipids in the NMR spectrum could be used to distinguish women with cancer from healthy subjects or from women with mammographical abnormalities due to benign disease. The line width correlated highly with the level of serum triclycerides. Correction for the level of triglycerides did not improve the diagnostic accuracy of the line width.

**Table IV** Regression model for cancer cases with line width as the dependent variable

| | *Parameter estimate (s.d.)* |
|---|---|
| Tumour size (mm) | 0.04 (0.08) |
| Tumour type (undifferentiated *vs* other) | 0.37 (1.0) |
| Nodal status (nodal metastases present *vs* absent) | 1.6 (1.2) |

Models corrected for age and triglycerides in continuous form.

The study design was rigorous and ideally suited to answer the question whether *in vitro* NMR spectroscopy can detect breast cancer in the preclinical stage. The study subjects comprised a consecutive sample from a population-based screening program. Almost all the patients with breast abnormalities were asymptomatic and the diagnosis of cancer in these cases was not established until after the operation – i.e., after the blood samples were drawn. Thus the patient's diagnosis was blind in relation not only to the NMR analysis but also to the person who drew the blood samples and took the history. We were able to adjust for the serum triglyceride level in every individual.

According to our results, NMR line width has virtually no power to detect early breast cancer. Although no one has systematically studied the domain of preclinical breast cancer, our results agree with those of several other investigators (Bell *et al.*, 1987; Nicholson & Nicholson, 1987; Small & Hamilton, 1987; Buchthal *et al.*, 1988; Berger *et al.*, 1989; Herring *et al.*, 1989; Engan *et al.*, 1990; Otvos *et al.*, 1991). No other study has included unselected controls from the same source population as the cases. Similar results which show that ROC analysis is of little diagnostic value have also been obtained by others (Otvos *et al.*, 1991). Two further reports (Peeling *et al.*, 1988; Höfeler & Scheulen, 1989) credit *in vitro* NMR spectroscopy with a greater capacity to detect cancer but they still found a considerable overlap between the various patient groups (Peeling *et al.*, 1988), and inconsistent results when the experiment was repeated (Höfeler & Scheulen, 1989). They did not determine the serum triglyceride levels (Peeling *et al.*, 1988; Höfeler & Scheulen, 1989).

We followed Fossel's laboratory methods carefully. The lack of a difference in line width in our investigation may be due to a low 'tumour burden' among the patients with cancer. This possibility is partially offset by the lack of correlation between a preoperative tumour burden and line width and by the lack of a significant change after removal of the primary tumour. Moreover, if the size of the breast cancer must be appreciably larger than in this study to be detected by NMR, this method would have little clinical value. The use of line width for distinguishing between benign and malignant disease would then fall short of other methods, such as mammography, cytology and clinical investigation.

In summary *in vitro* NMR spectroscopy in our hands was not a useful diagnostic tool in patients with early breast cancer.

The authors wish to thank Dr E. Thurfjell from the Mammography Screening Unit for kind assistance.

### References

BELL, J.D., SALDER, P.J., MACLEOD, A.F., TURNER, P.R. & LA VILLE, A. (1987). HNMR studies of human blood plasma. Assignments of resonances for lipoproteins. *FEBS Lett.*, **219**, 239–243.

BERGER, S., PFLÜGER, K.-H., ETZEL, W.E. & FISCHER, J. (1989). Detection of tumors with nuclear magnetic resonance properties of plasma. *Eur. J. Cancer Clin. Oncol.*, **25**, 535–543.

BUCHTHAL, S.D., HARDY, M.A. & BROWN, T.R. (1988). Assessing the value of identifying the presence of malignant disease in human plasma by proton NMR spectroscopy. *Am. J. Med.*, **85**, 528–532.

ENGAN, T., KRANE, J., KLEPP, O. & KVINNSLAND, S. (1990). Proton nuclear magnetic resonance spectroscopy of plasma from healthy subjects and patients with cancer. *N. Engl. J. Med.*, **322**, 949–953.

FOSSEL, E.T., CARR, J.M. & MCDONAGH, J. (1986). Detection of malignant tumors. Water suppressed proton nuclear magnetic resonance spectroscopy of plasma. *N. Engl. J. Med.*, **315**, 1369–1376.

HERRING, F.G., PHILIPHS, P.S. & PRITCHARD, P.H. (1989). Proton magnetic resonance spectroscopy of plasma from patients with dyslipoproteinemia: identification of factors governing methyl and methylene proton line widths. *J. Lipid Res.*, **30**, 521–528.

HÖFELER, H. & SCHEULEN, M.E. (1989). Monitoring of patients with non-seminomatous testicular cancer by nuclear magnetic resonance spectroscopy of plasma. *Eur. J. Cancer Clin. Oncol.*, **25**, 1141–1143.

MCNEIL, B.J. & HANLEY, J. (1984). Statistical approaches to the analysis of receiver-operating characteristics (ROC) curves. *Med. Decis. Making*, **4**, 137–150.

MIMS, M.P., MORRISETT, J.D., MATTIOLI, C.A. & GOTTO, A.M. (1989). Effect of triglyceride levels on methyl and methylene envelope line widths in proton nuclear magnetic resonance spectroscopy of human plasma. *N. Engl. J. Med.*, **320**, 1452–1457.

NICHOLSON, J.K. & NICHOLSON, F. (1987). Proton spectroscopy of plasma and testing for malignancy. *Lancet*, **II**, 280–281.

OKUNIEFF, P., ZIETMAN, A., KAHN, J., SINGER, S., NEURINGER, L.J., LEVINE, R.A. & EVANS, F.E. (1990). Lack of efficacy of water suppressed proton nuclear magnetic resonance spectroscopy of plasma for the detection of malignant tumors. *N. Engl. J. Med.*, **322**, 953–958.

OTVOS, J.D., JEYARAJAH, L., HAYES, W., FREEDMAN, D.S., JANJAN, N.A. & ANDERSON, T. (1991). Relationships between the proton nuclear magnetic resonance properties of plasma lipoproteins and cancer. *Clin. Chem.*, **37**, 369–376.

PEELING, J., SUTHERLAND, G., MARAT, K., TOMCHUK, E. & BOCK, E. (1988). $^{1}H$ and $^{13}C$ nuclear magnetic resonance studies of plasma from patients with primary intracranial neoplasms. *J. Neurosurg.*, **68**, 931–937.

SMALL, D.M. & HAMILTON, J.A. (1987). Correspondence. *N. Engl. J. Med.*, **316**, 1412–1413.

SWETS, J.A. (1988). Measuring the accuracy of diagnostic systems. *Science*, **240**, 1285–1292.

WILDING, P., SENIOR, M.B., INUBUSHI, T. & LUDWICH, M.L. (1988). Assessment of proton nuclear magnetic resonance spectroscopy for detection of malignancy. *Clin. Chem.*, **34**, 505–511.